# Continuous Theta Burst Stimulation of Angular Gyrus Reduces Subjective Recollection

**DOI:** 10.1371/journal.pone.0110414

**Published:** 2014-10-21

**Authors:** Yasemin Yazar, Zara M. Bergström, Jon S. Simons

**Affiliations:** 1 Department of Psychology, University of Cambridge, Cambridge, United Kingdom; 2 Behavioural and Clinical Neuroscience Institute, University of Cambridge, Cambridge, United Kingdom; 3 Department of Psychology, University of Kent, Kent, United Kingdom; University of California, Merced, United States of America

## Abstract

The contribution of lateral parietal regions such as the angular gyrus to human episodic memory has been the subject of much debate following widespread observations of left parietal activity in healthy volunteers during functional neuroimaging studies of memory retrieval. Patients with lateral parietal lesions are not amnesic, but recent evidence indicates that their memory abilities may not be entirely preserved. Whereas recollection appears intact when objective measures such as source accuracy are used, patients often exhibit reduced subjective confidence in their accurate recollections. When asked to recall autobiographical memories, they may produce spontaneous narratives that lack richness and specificity, but can remember specific details when prompted. Two distinct theoretical accounts have been proposed to explain these results: that the patients have a deficit in the bottom-up capturing of attention by retrieval output, or that they have an impairment in the subjective experience of recollection. The present study aimed to differentiate between these accounts using continuous theta burst stimulation (cTBS) in healthy participants to disrupt function of specific left parietal subregions, including angular gyrus. Inconsistent with predictions of the attentional theory, angular gyrus cTBS did not result in greater impairment of free recall than cued recall. Supporting predictions of the subjective recollection account, temporary disruption of angular gyrus was associated with highly accurate source recollection accuracy but a selective reduction in participants’ rated source confidence. The findings are consistent with a role for angular gyrus in the integration of memory features into a conscious representation that enables the subjective experience of remembering.

## Introduction

The left lateral parietal lobe consistently exhibits activity during neuroimaging studies of long-term memory retrieval [1]. However, patients with lateral parietal lesions are not amnesic, performing highly accurately on various memory tasks [2]–[4] even when lesions closely overlap with regions activated in healthy participants performing the same tasks [4]. Parietal lesion patients do show significantly reduced confidence when judging the context in which an item was previously encountered, with confidence in other aspects of memory unimpaired [5]. They also produce fewer subjective ‘remember’ responses on remember/know tasks [3], and exhibit diminished spontaneous recall of autobiographical memory details [2], [3]. One account of these findings is that patients with lateral parietal lesions may have a deficit in the bottom-up capturing of attention by retrieval output [6], [7], whereas another view is that patients’ subjective experience of recollection may be impaired, leaving objective recollection accuracy intact [5], [8], [9].

Differentiating between these competing theories is complicated by the functional heterogeneity within the lateral parietal lobe, with neuroimaging studies indicating numerous functionally distinct subregions [10], [11]. Recollection tends to be associated with the left angular gyrus (AnG), whereas familiarity-based memory is often linked with the more dorsal left intraparietal sulcus (IPS) [12], [13]. Establishing whether the pattern of memory performance after parietal lesions can be explained by impairments in bottom-up attention or subjective aspects of recollection is made difficult by technical limitations inherent to neuropsychological investigations. Patient lesions are rarely restricted to a single, circumscribed region, tending to involve both dorsal and ventral parietal areas, the amount of remote dysfunction (i.e., diaschisis) can be difficult to determine, and long-term brain damage may lead to functional reorganisation.

In the present study, we addressed these constraints using continuous theta burst stimulation (cTBS) [14] in healthy participants to disrupt function of specific left parietal subregions and examine patterns of impaired and preserved performance on an extensive battery of memory measures. The battery included tests of item recognition as well as recollection tasks such as free recall, cued recall, and source recollection, assessing both objective memory accuracy and subjective rated memory confidence. This approach enables the two competing theoretical accounts to be directly compared by examining performance across tasks within the same participants. Two key directional hypotheses were tested. If the attentional model is correct, and left AnG supports bottom-up attentional processes, then stimulation should lead to greater impairment on free recall, which is considered to rely predominantly on the bottom-up capturing of attention by mnemonic representations, than other measures of recollection such as cued recall, in which retrieval is thought to be guided more by external cues [2], [6]. In contrast, the subjective recollection view predicts intact recall and objective source accuracy after left AnG stimulation, but selectively reduced subjective confidence in source recollection, sparing other measures of confidence [5]. To address the question of anatomical specificity, AnG stimulation was compared with cTBS targeting the left IPS, and with a control site, the vertex.

## Materials and Methods

### Participants

75 healthy right-handed, native English speakers, aged 19–33 years (M = 24.57), were recruited from various volunteer panels in Cambridge. Participants were allocated to one of the three cTBS stimulation groups (AnG, IPS, vertex) in a sequential order. Data from 6 participants had to be excluded due to technical problems, leaving 23 subjects in each group who were matched for age, F (2, 66) = 1.13, n.s.), and gender, F (2, 66) = 0.76, n.s. All subjects had normal hearing and normal or corrected-to-normal vision and were screened for possible contra-indications to cTBS. Subjects gave their written informed consent to participate in the study which was approved by the University of Cambridge Human Biology Research Ethics Committee, and were reimbursed for their participation.

### Stimuli

Two hundred and fifty-six nouns were selected from the Medical Research Council Psycholinguistics database (http://tinyurl.com/mrc-database). All words were between three and eight letters long, with a Kucera-Francis written Frequency of 20–100 and concreteness and imageability ratings of 500–700. Words were randomly allocated to pairs. One hundred and twenty-eight words, i.e. 64 word pairs, were used in the study phase as “old” words, while the other half were added in the test phase as “new” words. All word stimuli were recorded by four different native English speakers (two female, two male) with the audio editor and recorder *Audacity*. Four different speakers recorded the words for the study and test phase so that the speaker identity always changed between study and test, with half the studied words spoken by a speaker of the same gender at test, and half spoken by a speaker of different gender. This assignment resulted in six possible combinations of old/new and female/male voice during the test phase and eight different counterbalancing formats that were rotated across participants. During the experiment, all spoken responses were recorded with *Audacity*.

### Procedure

Each participant underwent the same procedure: assessment of resting motor threshold, study phase, cTBS procedure, test phase. Upon arrival, each individual’s resting motor threshold was assessed by single-pulse stimulation (for details see ‘cTBS procedure’). The experiment started with the study phase: word pairs were auditorily presented via a headset with an attached microphone. Word-pairs were either spoken by a female voice referred to as ‘Olivia’ or by a male voice referred to as ‘George’, with no more than four consecutive trials read by the same person. A fixation cross was presented for 500 ms and remained on the screen while subjects listened to the two words. Subsequently, three question marks appeared on the screen for 10 seconds during which subjects were instructed to form a sentence that included both the heard words and had the speaker of the words, i.e. either George or Olivia, as a character in the sentence (e.g., “George’s sister lived in a valley”, with ‘sister’ and ‘valley’ being the study words that were spoken in a male voice). This procedure was repeated for all 64 word pairs.

Instructions for the memory test phase were then provided, followed by administration of cTBS. After the cTBS procedure, the experiment continued with a series of memory tasks (the delay between study and the first memory test was typically ∼25 mins). First, subjects were asked to freely recall as many of the previously studied words as they could remember within four minutes. Then a brief version of the memory test instructions was repeated before the subject performed computerized memory tasks that included old/new recognition, cued recall, and source recollection. Some number processing tasks were also administered shortly before stimulation, after stimulation, and at the end of the experiment, the results of which are not reported here.

**Figure 1:**
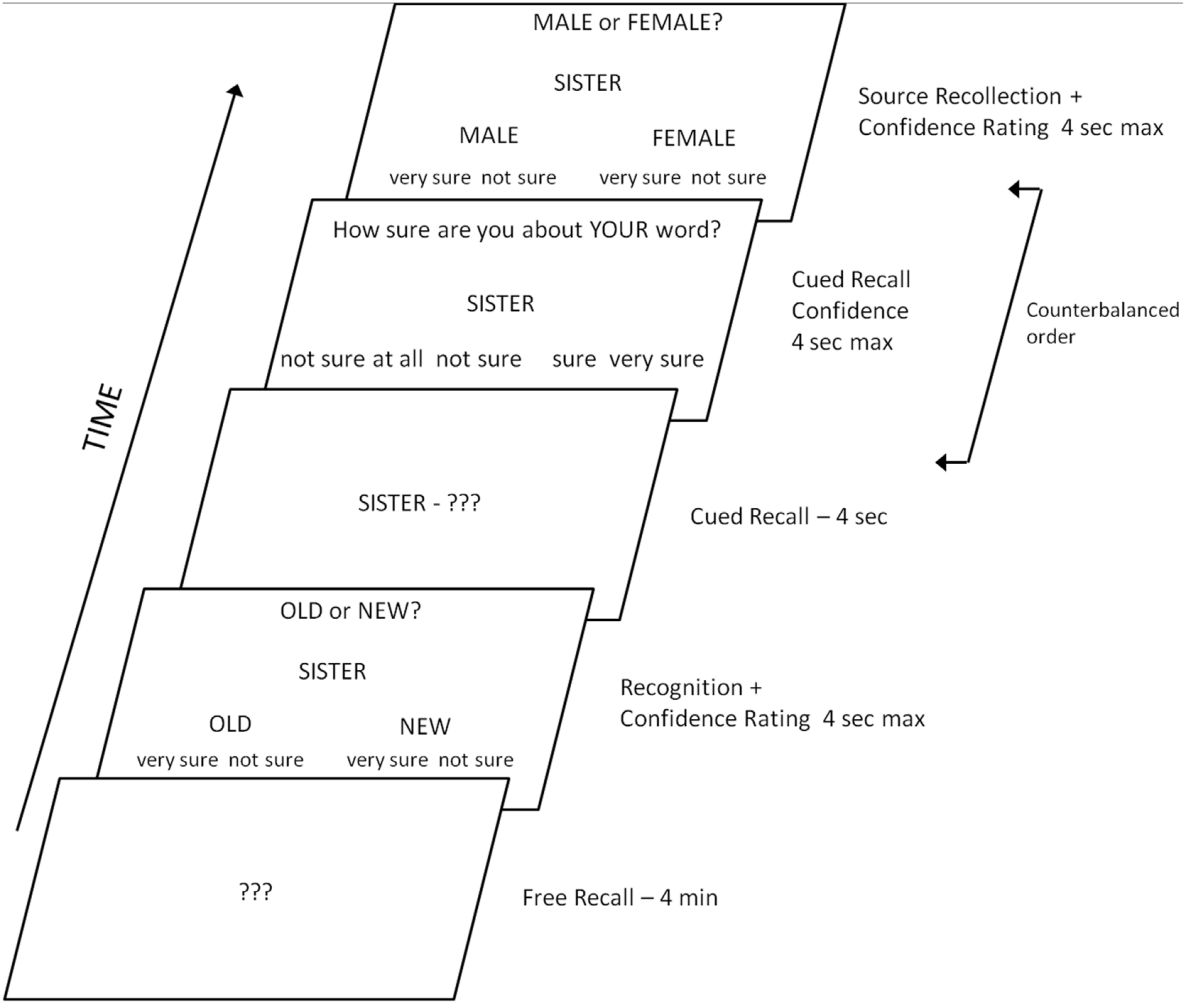
Schematic diagram illustrating the computerized memory tests.

At test, subjects heard single words over the headphones, half of which had been presented during study (old words) and half of which were new words. In addition, each word was also visually presented in the middle of the screen and remained there for all subsequent judgments in that trial. The test phase is illustrated in Figure 1. First, a screen prompted participants to make an old/new recognition judgment. The question “Old or New?” was displayed at the top of the screen, above the stimulus word. The response options ‘Old’ or ‘New’ were presented on the left and right side towards the bottom of the screen with the confidence judgment ‘very sure’ and ‘not sure’ displayed under each judgment. Throughout the test phase subjects used the middle and index finger of each hand to respond. The response buttons were the ‘z’, ‘x’, ‘n’, and ‘m’ keys on the keyboard. If subjects responded with one of the ‘New’ buttons, the next stimulus was presented. However, if the word was endorsed as old, cued recall and source recollection were tested. The order of presentation for these two memory tasks was counterbalanced across subjects.

In cued recall, subjects were asked to remember the associate word that had previously been presented along with the target word, and say it out loud. Subjects were instructed to make their best guess if they were unsure about an answer. Subsequently, they judged their confidence in their cued recall on a scale from ‘not sure at all’, ‘not sure’, ‘sure’, and ‘very sure’. The source recollection task prompted subjects to judge whether the target word had initially been spoken by the male or by the female speaker. The question “Male or Female?” was displayed at the top of the screen, above the stimulus word. The response options ‘Male’ or ‘Female’ were presented on the left and right side towards the bottom of the screen with the confidence judgment ‘very sure’ and ‘not sure’ displayed under each judgment. Subjects had four seconds for each judgment.

### cTBS procedure

**Figure 2:**
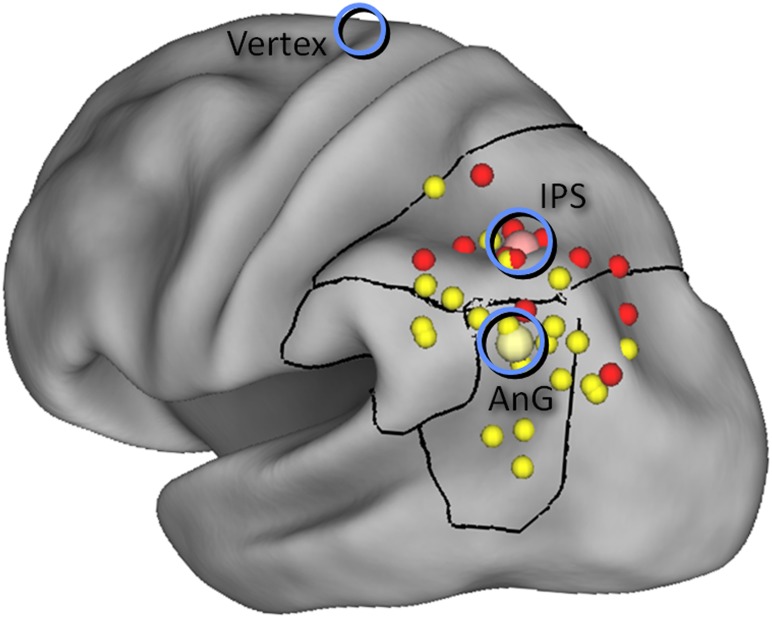
cTBS target locations (blue circles) displayed on an inflated fiducial brain that illustrates parietal loci sensitive to recollection (small yellow spheres) and familiarity (small red spheres), derived from a meta-analysis of fMRI studies [13]. The centers of mass of these activation clusters (large spheres) were the targets for angular gyrus (AnG) and intraparietal sulcus (IPS) stimulation. Figure adapted from one kindly provided by Mick Rugg.

At the beginning of each session, the subject’s individual resting motor threshold was assessed for the right first dorsal interosseous (FDI) hand muscle. After the study phase, each subject’s head was co-registered to their brain image via previously identified anatomical landmarks using the neuronavigation system software Brainsight (Rogue Research, Canada). To guide frameless stereotaxy we used target centre of mass MNI coordinates described in a previous meta-review of the parietal lobe and memory [13] for AnG and IPS: AnG (–43, −66, 38), IPS (–38, −62, 46), and a probabilistic anatomical atlas [15] for vertex (0, −15, 74), as illustrated in Figure 2. Then a standard conditioning cTBS protocol was delivered with three pulses at 50 Hz, repeated at 200 ms for 40 sec at 70% resting motor threshold to either left AnG, left IPS or vertex [14]. Stimulation was delivered via a Magstim Rapid^2^ (Whitland, UK) with a standard 70 mm diameter figure-of-eight coil.

## Results

Cued recall and source recollection were computed as a proportion of items endorsed as ‘old’ in the recognition test, source confidence was calculated for the trials that received correct source judgments. Mean recognition accuracy (hits minus false alarms), cued and free recall and source recollection accuracy and respective confidence values (proportion of trials receiving a ‘very sure’ response) are displayed in Table 1. Recognition accuracy calculated using d-prime was: vertex (M = 2.57, SD = 0.55), AnG (M = 2.69, SD = 0.51), IPS (M = 2.59, SD = 0.57).

**Table 1:** Performance of the three participant groups on the battery of memory tests.

	Vertex		AnG		IPS	
Memory measure	M	SD	M	SD	M	SD
Free recall	0.19	0.07	0.18	0.06	0.19	0.05
Old-new recognition	0.77	0.11	0.77	0.09	0.75	0.10
Recognition confidence						
Old items	0.79	0.09	0.81	0.10	0.75	0.12
New items	0.51	0.16	0.55	0.25	0.49	0.17
Cued recall	0.51	0.21	0.50	0.17	0.51	0.18
Cued recall confidence	0.52	0.17	0.55	0.20	0.52	0.18
Source recollection	0.81	0.12	0.77	0.15	0.79	0.14
Source confidence	0.65	0.15	0.55	0.22	0.63	0.19

Note: AnG = angular gyrus, IPS = intraparietal sulcus, M = mean, SD = standard deviation, old-new recognition calculated as hits minus false alarms, all other accuracy measures as proportion correct. Confidence values reflect the proportion of correct trials receiving a ‘very sure’ response.

**Figure 3:**
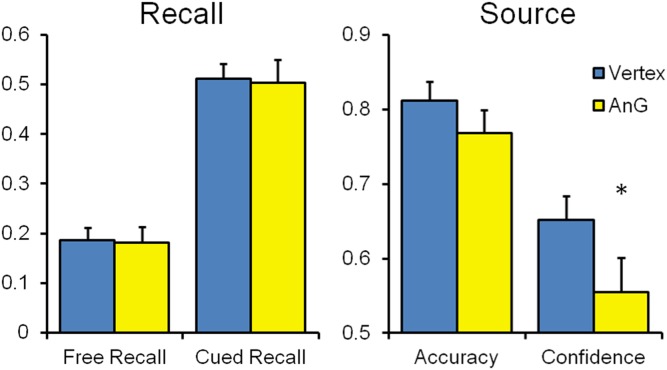
Performance of the groups who received cTBS targeting vertex and angular gyrus (AnG) on free and cued recall (left panel) and source recollection accuracy and confidence (right panel). Error bars denote standard error of the mean.

The competing attentional and subjective recollection accounts were tested by examining their a priori directional predictions using paired- and independent-samples t-tests as appropriate, with one-tailed alpha set at 0.05. One-tailed tests are applicable in this case because we are testing only for the presence of cTBS-induced impairments in performance. Effect sizes were computed using Cohen’s d. The main prediction of the attentional model was that AnG stimulation would impair free recall but not cued recall, because free recall relies more on the bottom-up capturing of attention by mnemonic representations. Consistent with the more attentionally demanding nature of free recall, all three participant groups produced fewer correct free recall than cued recall responses, all t (22)>9.03, p<0.001, d>1.48. However, as displayed in Figure 3, when comparing performance of the AnG and vertex groups directly, the results revealed that free recall was entirely unimpaired following stimulation of AnG compared with vertex, t (44) = 0.24, n.s., d = 0.07, as was cued recall, t (44) = 0.127, n.s., d = 0.04. Performance of all groups on free recall was well above floor levels, all t (22)>12.66, p<0.001, d>2.64, precluding a possible task difficulty explanation for the observed results.

The findings of intact free recall and cued recall following AnG stimulation do not support the attentional model but are consistent with the subjective recollection perspective. The main prediction of this alternative view was that objective source accuracy would be unaffected by AnG stimulation, but that selectively reduced subjective confidence in source recollection would be observed. As predicted, AnG stimulation did not significantly reduce source accuracy, t (44) = 1.10, n.s., d = 0.33, but source recollection confidence in the AnG group was significantly diminished as compared to the vertex group, t (44) = 1.74, p<0.05, d = 0.52 (Figure 3). To evaluate this effect, the probability that a randomly selected individual from the AnG group would have lower source confidence than a randomly selected person from the vertex group was 64% [16].

The confidence reduction after AnG stimulation was specific to source confidence. Confidence of participants from the AnG group in their correct recognition of old items was significantly higher than their confidence in their correct source recollection of those items, t (22) = 5.24, p<0.001, d = 1.42, a finding that cannot be attributed to differences in difficulty because old-new and source accuracy were very similar, t (22) = 0.12, n.s., d = 0.03. Unimpaired recognition confidence in the AnG group compared to the vertex group was observed similarly for both old items, t (44) = 0.70, n.s., d = 0.21, and new items, t (44) = 0.68, n.s., d = 0.21. Similarly, cued recall confidence was preserved, t (44) = 0.46, n.s., d = 0.14.

There was some evidence that the decrease in source confidence may be anatomically selective to AnG. In contrast to the clear reduction exhibited by the AnG group, source confidence ratings of the vertex and IPS groups were virtually identical, t (44) = 0.50, n.s., d = 0.15. When comparing the AnG and IPS groups directly, the diminished recollection confidence in the AnG group did not reach significance (p = 0.125), t (44) = 1.17, d = 0.35. There was, however, a 59% probability that a randomly selected person from the AnG group would have lower source confidence than a randomly selected participant from the IPS group. It should be noted that the AnG and IPS target regions derived from Vilberg & Rugg’s (2008) meta-analysis of fMRI studies were only 10.3 mm apart, a proximity that may be right at the limit of the spatial resolution of cTBS [17].

## Discussion

In the present study, we identified a causal relationship between the AnG sub-region of the left lateral parietal lobe and the subjective experience of recollection, whereby temporarily disrupting the left AnG with continuous theta burst stimulation (cTBS) diminished participants’ rated confidence in their accurate source recollection judgments. This recollection confidence reduction contrasted with unimpaired objective memory accuracy in the same group, demonstrating a specific link between the AnG and individuals’ subjective recollection. Notably, AnG stimulation had no effect on participants’ free recall or cued recall, arguing against an account of the region’s function in terms of the bottom-up capturing of attention by retrieval output.

The results converge with previous neuropsychological studies suggesting that the lateral parietal lobe is not necessary for accurate recollection, since parietal lesion patients show intact performance on tasks that measure objective aspects of memory, such as recognition [3], [5], [8], [18], source accuracy [3]–[5], and cued recall [3]. Our findings are also consistent with neuropsychological evidence across three separate experiments that parietal lesion patients selectively report significantly reduced confidence in their source recollection judgments [5], indicating that their personal experience of a recollected memory, such as the richness and vividness of its episodic detail, is impaired. Note that the confidence reduction following AnG stimulation was specific to source recollection, with recognition and cued recall confidence unimpaired, consistent with the parietal lesion data which has also been characterized by source-specific confidence reductions [5], suggesting that the observed effects do not merely reflect reduced confidence in participants’ cognitive abilities overall.

The intact levels of confidence for recognition and cued recall of word-pairs observed in the present data are consistent with evidence that old-new item discrimination and cued recall of within-modality paired associations may be able to be accomplished largely on the basis of familiarity processes [19], whereas recollection is required for the retrieval of integrated word-voice context links as in the present source memory task [20]. Alternatively, if word cue recall is considered predominantly reliant on retrieval of semantic features whereas source memory for voice is largely dependent on retrieval of perceptual features [20], then it may be that AnG is more important for confidence judgments about perceptual than semantic features. However, this latter possibility seems inconsistent with the literature identifying AnG as a key region for semantic processing [21], and with the patient study by Berryhill et al. [2] that tested autobiographical memory, observing a lack of richness and specificity in both episodic and semantic aspects of parietal lesion patients' recollections (although see Davidson et al. [3] for evidence of spared memory for semantic elements of autobiographical events). In any case, the current experiment extends the patient literature by demonstrating that disruption of AnG function may be specifically responsible for the observed decreases in subjective aspects of recollection such as source confidence, autobiographical recall, and ‘remember’ judgments [2], [3], [5], establishing the causal link with greater anatomical selectivity than was previously possible based on neuropsychological findings alone. However, it should be borne in mind that despite the relatively large number of participants in the current study, it was only possible to demonstrate a numerical (13%), but not significant, difference in source confidence between the anatomically-proximal AnG and IPS groups, perhaps reflecting technical limitations in the spatial resolution of cTBS.

Whereas the present results provide support for the subjective recollection account of AnG function, the observation that free recall was just as unaffected by AnG stimulation as cued recall is inconsistent with the main prediction of the alternative attention to memory hypothesis. According to this view, left ventral parietal regions including the AnG subserve bottom-up attentional processes that are captured by behaviorally-relevant information retrieved from memory [6]. The spontaneous free recall of studied words would seem to be be an archetypal task for recruiting such attentional processes, relying, it is argued [2], [6], considerably more than tasks such as cued recall or source recollection on memories popping out automatically and capturing attention. The present results align with evidence from a number of previous studies in questioning predictions of the attention to memory model. For example, patients with parietal lesions exhibited no disproportionate impairment in a recollection task that manipulated the behavioral relevance of information that was to be retrieved from memory [5]. Similarly, although one study reported that parietal patients responded more slowly in a memory analog of the Posner cueing task in which anticipatory cues preceded each memory test trial [7], the same pattern of results could not be replicated subsequently with a similar task [18]. Perhaps most compellingly, Hutchinson et al. [12] noted that whereas recollection effects are typically observed in the left AnG region that was targeted in the present study [13], bottom-up attention is generally associated with the more anterior temporoparietal junction area in the right hemisphere [22]. Evidence linking this right hemisphere area with bottom-up influences on memory (rather than the left parietal region specified in the attention to memory model) comes from recent fMRI data indicating that unintentional recall of memories that participants tried to suppress but which nevertheless came to mind was associated with activity in right ventral parietal cortex [23]. It may be that the attention to memory model might be useful in guiding hypotheses about right parietal contributions to memory.

The results of the present study instead corroborate the hypothesis that the left AnG mediates the subjective experience of contextual recollection. In support of this view, previous fMRI studies have reported left inferior parietal cortex activity that was greater during subjective memory assessments (e.g., confidence ratings) than objective memory decisions (e.g., source judgments) [24]–[26]. In addition, a recent TMS study by Sestieri et al. [27] found that rTMS targeting the left AnG affected participants’ response bias when making source memory attributions, which was interpreted as indicating a role for this region in subjective aspects of source monitoring associated with the weighing of relevant retrieved information. The study by Sestieri et al. found no effect on objective or subjective memory of stimulating a more dorsal parietal region around the IPS, consistent with the results of the present experiment. Similarly, Rossi et al. [28] reported that retrieval was unimpaired after rTMS targeting IPS. This lack of sensitivity to neurostimulation of the IPS across studies is difficult to account for. The cTBS target locations in the present experiment were based on centre of mass coordinates from a meta-review of fMRI studies of memory by Vilberg and Rugg [13], so it may be fruitful for future studies to target stimulation based more on individual anatomical landmarks. Having said that, measuring the distance for each participant in the current study between the site of stimulation and her/his actual IPS did not indicate that the targeting method was likely to have made a difference to the results observed. Such methodological difficulties do not seem to be as much of a concern for AnG, where the stimulation site (also derived from Vilberg and Rugg’s meta-review) was sufficiently sensitive to cTBS to elicit a source recollection confidence reduction that had an effect size above 0.5.

Accruing evidence thus points towards a causal role for the AnG in subjective elements of recollection, but the information processing operations subserved by this region that lead to the qualitative experience of “re-living” the past remain unclear [9]. One possibility is that the AnG is involved in integrating modality-specific memory features distributed in other cortical regions into a multimodal conscious representation that enables the rich and vivid subjective “re-living” of an event with all its attendant sights, sounds, and smells. As noted above, the region has previously been considered a hub for multisensory integration in other domains, such as semantic memory [21], [29], and it may be that during episodic memory retrieval, AnG provides the multisensory binding that creates a coherent and vivid conscious experience by integrating event-specific features retrieved from memory. A similar idea has been put forward as the cortical binding of relational activity theory [30], but that account emphasizes a post-consolidation role for the lateral parietal lobe in supporting binding during retrieval of consolidated episodic memories that are no longer bound by the hippocampus. Such a distinction would appear to be inconsistent with the numerous effects that have been observed, as in the present study, for stimuli that were studied only a number of minutes before test, presumably well before consolidation can be expected to have occurred. Our alternative multisensory integration account suggests that the AnG may be particularly important for the retrieval of memory traces that involve several different types of features (sensory, conceptual, internally generated, etc.). This putative combinatory role can be differentiated from the pattern completion binding processes associated with the hippocampus [31], [32] in that the AnG may integrate mnemonic features within an egocentric rather than allocentric framework [33], [34], enabling the first-person perspective re-experiencing of a past event that is such a cardinal feature of episodic memory [35].

If this is the case, it is possible that with the right tasks, it may be possible to demonstrate that the AnG is important for some aspects of objective recollection performance. Future studies could test the effect of parietal disruption on memory tests that are more sensitive to the quality of information recollected, for example by testing multi-dimensional source memory judgments. A further desirable avenue for future research is to investigate the effects of temporary disruption to the AnG in both hemispheres. The present study employed only left lateral parietal stimulation because neuroimaging research has shown predominantly left-lateralised parietal activations during memory retrieval [1], [13], and theories that have been proposed to account for these effects have emphasized the role of left lateral parietal regions (e.g., [6]). However, some patients who show reduced subjective quality of recollection have bilateral damage [2], [5], and so for a full understanding of the role of the lateral parietal lobe in episodic retrieval it will be important to explore potential laterality effects.

In conclusion, the current experiment investigated the effect on different measures of objective and subjective memory of stimulating sub-regions of the lateral parietal lobe. We found a specific causal relationship between the AnG and source recollection confidence, consistent with a role in the integration of memory features into a conscious representation that enables the subjective experience of remembering.
